# Effect of dapagliflozin on left ventricular structure and function in patients with non-ischemic dilated cardiomyopathy: An observational study

**DOI:** 10.1097/MD.0000000000037579

**Published:** 2024-03-29

**Authors:** Jun Hong, Lei Huang, Nake Jin, Xuechen Zhao, Jianan Hu

**Affiliations:** a Department of Cardiology, Ningbo Hangzhou Bay Hospital, Ningbo, China.

**Keywords:** cardiac function, dapagliflozin, dilated cardiomyopathy, non-ischemic dilated cardiomyopathy, sodium glucose cotransporter type 2 inhibitors

## Abstract

Non-ischemic dilated cardiomyopathy (NIDCM) is characterized by left ventricular dilatation and contractile dysfunction with severe morbidity and mortality. Sodium glucose cotransporter type 2 (SGLT2) inhibitors significantly reduce cardiovascular events for heart failure patients. We performed to investigate the impact of combined administration of SGLT2 inhibitors on cardiac structure and function in NIDCM patients undergoing conventional therapy. A total of 50 newly diagnosed NIDCM patients received conventional medical therapy, with 23 receiving dapagliflozin 10mg/day in addition (SGLT2i group) and the remaining 27 only receiving conventional therapy (non-SGLT2i group). After 12 months outpatient follow-up, NIDCM patients treated with conventional therapy alone showed a significant reduction of left ventricular end-diastolic dimensions (LVEDd), left ventricular end-systolic dimensions (LVESd), left ventricular end-diastolic volumes (LVEDV), left ventricular end-systolic volumes (LVESV), left ventricular end-diastolic volume index (LVEDVi) and left ventricular end-systolic volume index (LVESVi), while an increase in fractional shortening (FS) and left ventricular ejection fraction (LVEF). Patients receiving dapagliflozin combined with conventional treatment also demonstrated a significant reduction in left ventricular dimensions and volumes, and a marked increase in cardiac function. In non-SGLT2i groups, the % change in LVEDd, LVESd, LVEDV, LVESV, LVEDVi, LVESVi, FS and LVEF was −2.8%, −4.6%, −6.2%, −10.1%, −6.1%, −10.1%, +9.7%, +11%. A greater absolute % fall in left ventricular volume in SGLT2i groups compared to non-SGLT2i groups resulted in a significant improvement in cardiac function. The results showed that SGLT2i combined with conventional therapy has a better beneficial effect on left ventricular volumes and cardiac function in NIDCM patients.

## 1. Introduction

Dilated cardiomyopathy (DCM) is defined as a serious heart disease with significant morbidity and mortality due to complications such as heart failure (HF) and arrhythmias.^[1]^ Adverse anatomical left ventricular (LV) remodeling, reflected by the presence of LV volumes increased and contractility decreased, is at the core of the pathophysiology of DCM, and the extent of adverse LV remodeling correlates with risks of hospitalization and cardiovascular death.^[2]^ Epidemiological studies have shown that the prevalence of DCM was estimated to be approximately 1 in 250 individuals,^[3]^ so the management and treatment of DCM are particularly important. Congestive HF is the most common symptom of DCM. Patients with low left ventricular ejection fraction (LVEF) or severe diastolic dysfunction have the worst prognosis. Therefore, medical treatment of DCM, as with medications for heart failure, aims to be significant clinical benefits such as improved survival and reduce rehospitalization rates, including angiotensin receptor-neprilysin inhibitor, angiotensin-converting enzyme inhibitors (ACEi), angiotensin receptor blockers (ARB), beta-adrenoreceptor blockers (BB), aldosterone antagonists.^[4]^ Despite the extensive phenotype and complex pathophysiology of DCM, ischemic heart disease remains the predominant cause. From a practical perspective, non-ischemic dilated cardiomyopathy (NIDCM) is a relatively prevalent form of DCM characterized by chamber enlargement and systolic dysfunction in the absence of coronary artery disease.^[5]^

The diabetic population in China is projected to experience a rapid increase, leading to a significant rise in the prevalence of cardiovascular disease among diabetic patients. Implementing appropriate interventions has the potential to effectively delay this progression.^[6]^ Sodium glucose cotransporter type 2 (SGLT2) inhibitors represent a new class of antidiabetic medications for the treatment of diabetes, and act inhibits renal glucose reabsorption, thereby increasing urinary glucose excretion and lowering the glucose burden on the organism.^[7–9]^ Since SGLT2 inhibitors have been extensively studied and demonstrated to significantly reduce cardiovascular events such as HF and myocardial infarction.^[10]^ The large clinical trials found that treatment with SGLT2 inhibitors for HF patients resulted in a lower rate of hospitalization or cardiovascular death.^[11]^ Scientific investigation has also shown that SGLT2 inhibitors can reduce the risk of arrhythmias.^[10]^

With the development of research, some experimental studies have explored SGLT2 inhibitors for the treatment of DCM. Animal experiments confirm that treatment with dapagliflozin in the doxorubicin-induced DCM mice model significantly improved the depressed LVEF and fractional shortening (FS) and decreased left ventricular internal dimensions at end-diastole (LVIDd) and end-systole (LVIDs).^[12]^ The clinical practice of patients with DCM treated with SGLT2 inhibitors are not well validated. Only a few studies with limited patient numbers have assessed the role of SGLT2 inhibitors in patients with DCM and NIDCM.^[13]^ The purpose of this study was to further explore the effect of the combined use of SGLT2 inhibitors on cardiac structure and systolic function in patients with newly diagnosed NIDCM treated with conventional therapy.

## 2. Materials and methods

### 2.1. Study population

The inclusion criteria were defined as follows: patients with New York Heart Association (NYHA) functional class II to IV and impaired LVEF of 40% or less, accompanied by cardiac dilatation confirmed through transthoracic echocardiography within 1 year; the absence of significant coronary artery disease, defined as luminal stenosis of at least 50% in one or more major coronary arteries, confirmed by either invasive coronary angiography or CT coronary angiography before study enrollment; regularly followed-up in the outpatient department. Exclusion criteria comprised of: heart failure resulting from other cardiomyopathies or diseases (such as valvular disease, congenital heart disease, tachycardia-induced cardiomyopathy, hypertensive cardiomyopathy, alcohol-related heart disease and stress-related heart disease); type 1 diabetes mellitus; estimated glomerular filtration rate less than 30 mL/min/1.73 m^2^; systolic blood pressure (BP) below 90 mm Hg; stroke; acute myocardial infarction; unstable angina; uncorrected valvular heart disease; hepatic disease (serum transaminase greater than 3times normal); drug abuse; or any other life-threatening noncardiac disease.

From October 2020 to November 2021, a total of 50 participants were enrolled in this study. All patients presented with heart failure symptoms upon admission, which were relieved by conventional treatment included ARNi/ (ACEi)/ARB, BB, aldosterone antagonists, and diuretics. Among them, 23 NIDCM patients received dapagliflozin 10 mg/d in addition to conventional therapy (SGLT2i group), 27 NIDCM patients only received conventional therapy (non-SGLT2i group). After being discharged from the hospital, the follow-up period commenced. Outpatients were subsequently monitored in specialized clinics through regular visits. The trans-thoracic echocardiogram was performed at baseline and after 12 months of treatment.

All patients underwent baseline investigations. Five mL of venous blood from the antecubital vein was drawn in the morning after an overnight fasting condition and then sent blood samples to the laboratory center to detect a complete hemogram, renal and liver function test, blood sugar, lipid profile, and BNP estimation.

### 2.2. Echocardiography

According to the consistent protocol, an independent professional in cardiology conducted all the testing of transthoracic echocardiography. All patients underwent transthoracic echocardiography using Philips EPIQ 7C equipment with an X5 transducer while in the left lateral decubitus position. A variety of 2D echocardiographic and doppler indices were recorded, including left ventricular end-diastolic dimensions and volumes (LVEDd, LVEDV), left ventricular end-systolic dimensions and volumes (LVESd, LVESV), LVEF, and FS. The left ventricular end-diastolic volume index (LVEDVi) and left ventricular end-systolic volume index (LVESVi) were calculated as LVEDV or LVESV divided by body surface area. All patients underwent repeat assessment at 12 months of follow-up.

### 2.3. Statistical analysis

The baseline characteristics of the participants were stratified into non-SGLT2i and SGLT2i groups. The median (interquartile range) was used for continuous variables, while categorical variables were expressed in numbers and percentages. Statistical comparisons between the 2 groups were performed using χ^2^ tests for categorical variables, one-way ANOVA tests for normally distributed data, or Wilcoxon-Mann-Whitney tests for non-normally distributed data. A multivariate analysis of covariance was performed using a linear combination of the change of echocardiographic indices as the dependent variable. The change of echocardiographic parameters was used as dependent variables: LVEDd, LVESd, LVEDV, LVESV, LVEDVi, LVESVi, FS, and LVEF. Age, Gender, BMI, heart rate, SBP, and DBP were used as covariates to control for individual differences. *P* value < .05 was considered significant. Data analysis was performed using IBM SPSS statistical software version 20.

## 3. Results

Both baseline and 12-month outpatient follow-up echocardiography data were available for 27 NIDCM in the non-SGLT2i group and 23 NIDCM in the SGLT2i group. Table 1 summarizes the demographics and baseline characteristics of the participants. The majority of participants were receiving guideline-recommended medications that improve cardiovascular prognoses, including ARNi/ACEi/ARB, BB, and aldosterone antagonists.

**Table 1:** Baseline characteristics of patients.

	Non-SGLT2i (n = 27)	SGLT2i (n = 23)	*P*
Males, n (%)	21 (77.8)	19 (82.6)	.670
Age, years	67 (59,74)	65 (51,72)	.302
Comorbidities			
Atherosclerosis of coronary artery, n (%)	11 (40.7)	7 (30.4)	.449
Hypertension, n (%)	13 (48.1)	10 (43.5)	.741
Diabetes, n (%)	10 (37.0)	13 (56.5)	.168
Dyslipidemia, n (%)	12 (44.4)	12 (52.2)	.586
Atrial fibrillation, n (%)	3 (11.1)	6 (26.1)	.170
BMI, kg/m^2^	23.94 ± 4.85	24.47 ± 3.86	.680
Heart rate, bpm	76.08 ± 11.53	83.17 ± 17.14	.218
Systolic blood pressure, mm Hg	128 (109,145)	116 (102,135)	.288
Diastolic blood pressure, mm Hg	74 (63,86)	78 (60,90)	.418
HbA1c, %	6.0 (5.4,6.3)	6.5 (5.9,7.5)	.043
Glucose, mmol/L	5.69 (4.84,6.38)	6.61 (5.58,7.50)	.025
Total cholesterol, mmol/L	3.96 (3.49,4.77)	4.21 (3.50,5.40)	.843
LDL-C, mmol/L	2.20 (1.84,2.60)	2.40 (2.06,3.35)	.387
HDL-C, mmol/L	1.16 (0.97,1.36)	1.04 (0.84,1.30)	.210
Triglyceride, mmol/L	1.24 (0.94,1.73)	1.44 (0.86,1.95)	.892
Creatinine, μmol/L	85.00 (71.00,100.44)	93.54 (86.45,111.56)	.090
Uric acid, μmol/L	385.00 (339.00,444.00)	457.00 (306.00,581.00)	.210
Hemoglobin, g/L	134.00 (124.00,154.00)	136.00 (123.50,157.25)	.599
NT-proBNP, pg/mL	840.00 (415.49,1868.58)	693.29 (373.50,2168.52)	.823
NYHA functional class			0.193
II	4 (14.8)	5 (21.7)	
III	19 (70.4)	10 (43.5)	
IV	4 (14.8)	8 (34.8)	
Cardiac implantable electronic devices, n (%)	1 (3.70)	1 (4.35)	.908
Medications, n (%)			
ARNI/ACEi/ARB	25 (92.6)	21 (91.3)	.857
BB	22 (81.5)	18 (78.3)	.777
Diuretic agents			
Other diuretic agents	23 (85.2)	18 (78.3)	.525
Aldosterone antagonists	25 (92.6)	20 (87.0)	.508
Oral anticoagulant	3 (11.1)	6 (26.1)	.170
Statin	12 (44.4)	12 (52.2)	.586
ASA/P2Y_12_ inhibitor	11 (40.7)	7 (30.4)	.449
Oral hypoglycemics	6 (22.2)	8 (34.8)	.324
Insulin	2 (7.4)	4 (17.4)	.279

ACEi = angiotensin-converting enzyme inhibitors, ANRi = angiotensin receptor-neprilysin inhibitor, ARB = angiotensin receptor blockers, ASA = acetylsalicylic acid, BB = beta-adrenoreceptor blockers, BMI = body mass index, HbA1c = glycated hemoglobin, HDL-C = highdensity lipoprotein cholesterol, LDL-C = low density lipoprotein cholesterol.

There was no significant difference between the 2 groups in terms of baseline echocardiographic parameters. After 12 months outpatient follow-up, compared to data between baseline and second examination, NIDCM patients treated with conventional therapy alone showed a significant reduction of LVEDd (68.30 ± 6.83 to 66.37 ± 6.90 mm), LVESd (57.15 ± 6.49 to 54.52 ± 6.48 mm), LVEDV(244.7 ± 54.62 to 229.61 ± 52.77 mL), LVESV (163.77 ± 42.6 to 147.21 ± 39.36 mL), LVEDVi (132.47 ± 31.64 to 124.39 ± 30.26 mL/m^2^), and LVESVi (88.65 ± 23.87 to 79.68 ± 22.09 mL/m^2^), while an increase in FS (16.31 ± 4.83 to 17.90 ± 4.08%) and LVEF (31.99 ± 8.49 to 35.52 ± 7.46%). Patients receiving dapagliflozin combined with conventional therapy also demonstrated a significant reduction in LVEDd (64.3 ± 7.99 to 59.35 ± 8.08 mm), LVESd (53.61 ± 9.46 to 47.39 ± 9.09 mm), LVEDV (214.98 ± 62.99 to 179.83 ± 56.26 mL), LVESV (144.78 ± 59.32 to 109.69 ± 49.29 mL), LVEDVi (111.62 ± 28.96 to 93.44 ± 26.08 mL/m^2^), and LVESVi (75.21 ± 28.16 to 56.81 ± 23.18 mL/m^2^), and a marked increase in FS(16.97 ± 7.23 to 20.63 ± 5.88%) and LVEF(27.87 ± 6.14 to 42.22 ± 9.88%). Echocardiography results of the SGLT2i and non-SGLT2i groups are shown in Table 2.

**Table 2:** Changes of echocardiogram results in patients with or without SGLT2i.

	Non-SGLT2i				SGLT2i			
	Baseline	Second examination	Δ Change from baseline	*P* *	Baseline	Second examination	Δ Change from baseline	*P* *
LA diameter	49.37 ± 6.17	48.74 ± 5.02	−0.63 ± 3.50	.36	46.96 ± 6.49	46.09 ± 7.61	−0.64 ± 8.7	.74
IVST (mm)	9.63 ± 1.74	9.59 ± 1.67	−0.04 ± 0.98	.85	9.78 ± 1.48	10.00 ± 1.57	0.22 ± 1.41	.47
PWT (mm)	9.04 ± 1.60	9.33 ± 1.30	0.30 ± 1.41	.29	9.17 ± 1.27	9.43 ± 1.47	0.26 ± 1.32	.35
LVEDd (mm)	68.30 ± 6.83	66.37 ± 6.90	−1.93 ± 3.57	.01	64.30 ± 7.99	59.35 ± 8.08	−4.96 ± 3.76	<.01
LVESd (mm)	57.15 ± 6.49	54.52 ± 6.48	−2.63 ± 3.39	<.01	53.61 ± 9.46	47.39 ± 9.09	−6.22 ± 4.01	<.01
LVEDV (mL)	244.7 ± 54.62	229.61 ± 52.77	−15.09 ± 27.37	.01	214.98 ± 62.99	179.83 ± 56.26	−35.15 ± 27.92	< 0.01
LVESV (mL)	163.77 ± 42.6	147.21 ± 39.36	−16.55 ± 23.19	<.01	144.78 ± 59.32	109.69 ± 49.29	−35.08 ± 25.23	<.01
LVEDVi (mL/m^2^)	132.47 ± 31.64	124.39 ± 30.26	−8.08 ± 14.56	.01	111.62 ± 28.96	93.44 ± 26.08	−18.18 ± 14.30	<.01
LVESVi (mL/m^2^)	88.65 ± 23.87	79.68 ± 22.09	−8.97 ± 12.57	<.01	75.21 ± 28.16	56.81 ± 23.18	−18.40 ± 13.01	<.01
FS (%)	16.31 ± 4.83	17.90 ± 4.08	1.59 ± 3.41	.02	16.97 ± 7.23	20.63 ± 5.88	3.66 ± 6.07	.01
LVEF (%)	31.99 ± 8.49	35.52 ± 7.46	3.52 ± 4.01	<.01	27.87 ± 6.14	42.22 ± 9.88	14.35 ± 7.54	<.01

FS = fractional shortening, IVST = interventricular septum thickness, LA = left atrial, LVEDd = left ventricular end-diastolic diameter, LVEDV = left ventricular end-diastolic volume, LVEDVi = left ventricular end-diastolic volume indexed to body surface area, LVEF = left ventricular ejection fraction, LVESd = left ventricular end-systolic diameter, LVESV = left ventricular end-systolic volume, LVESVi = left ventricular end-systolic volume indexed to body surface area, PWT = posterior wall thickness. Δ delta (second examination-baseline).

* *P* values in the difference of values between baseline and second examination.

**Figure 1.**
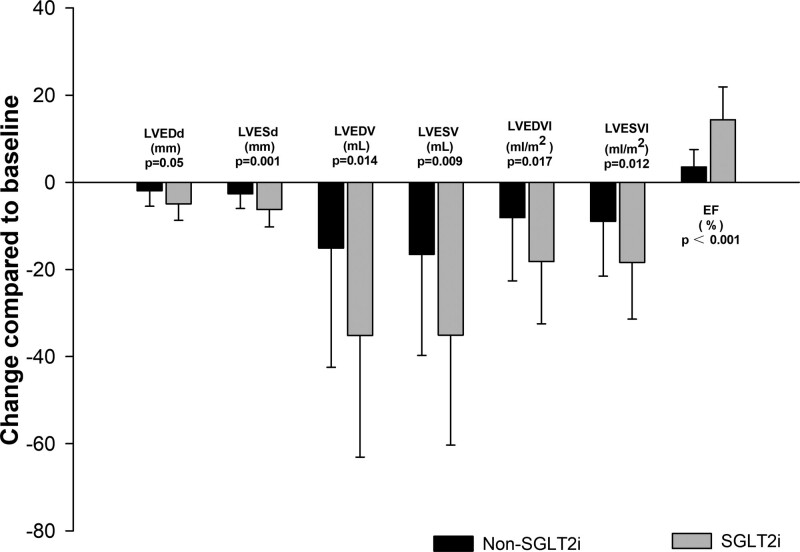
Change in secondary echocardiography outcomes from baseline to month 12.

There was a significant difference in the change of echocardiographic variables (LVEDd, LVESd, LVEDV, LVESV, LVEDVi, LVESVi, and LVEF) between the non-SGLT2i and SGLT2i groups (Table 2). Using multivariate analysis of covariance, it is evident that there exists a significant correlation between the improvement of echocardiographic parameters and the administration of SGLT-2 inhibitors (*P* = .001, Table S1, Supplemental Digital Content, http://links.lww.com/MD/L964). Figure 1 showed that SGLT2i combined with conventional therapy has a better beneficial effect on left ventricular volumes and on left ventricular ejection fraction in NIDCM patients. In non-SGLT2i groups, the % change in LVEDd, LVESd, LVEDV, LVESV, LVEDVi, LVESVi, FS, and LVEF was −2.8%, −4.6%, −6.2%, −10.1%, −6.1%, −10.1%, +9.7%, +11% respectively. A greater absolute % fall in LV volume in SGLT2i groups compared to non-SGLT2i groups resulted in a significant improvement in cardiac function (FS + 21.6%, LVEF + 51.5%).

## 4. Discussion

In this study, we further explore the effect of the combined use of SGLT2 inhibitors on LV structure and function in patients with NIDCM treated with conventional therapy. According to our study, by 12 months of follow-up, patients had a significant reduction in LV dimensions and volumes along with an improvement in cardiac function. The change in LV dimensions and volumes and cardiac function in NIDCM patients treated with conventional therapy were significant statistical differences after 12 months and these effects were progressively enhanced as patients received SGLT2i added to standard medical therapy.

DCM, including NIDCM, is the major cause of HF and has a poor prognosis. The pathophysiology of DCM is primarily characterized by adverse anatomical remodeling of LV, manifested by increased LV volumes and decreased contractility. Harmful LV remodeling is a characteristic feature of HF with reduced ejection fraction (HFrEF). The extent of adverse remodeling is directly associated with the risk of hospitalization and mortality.^[14]^ Additionally, the LVEF plays a crucial role in evaluating the severity of impaired cardiac systolic function and guiding the management of diverse cardiovascular disorders.^[15]^ A meta-analysis study suggest that the use of SGLT2 inhibitors resulted in significant improvements in LV regression, including LV mass (LVM), LVEF, LVEDV and LVESV. Additionally, there were notable enhancements in LV diastolic function, specifically the mitral inflow E velocity to tissue Doppler e’ ratio (E/e’) and left atrial volume index (LAVI). Subgroup analysis further confirmed that these improvements were primarily observed in patients with heart failure or those receiving empagliflozin treatment.^[14]^ Our study specifically investigated the impact of SGLT2 inhibitors on left ventricular structure and function in patients diagnosed with NIDCM. The results demonstrated that the combination of SGLT2 inhibitors and conventional therapy exhibited superior efficacy in enhancing left ventricular volume and function among NIDCM patients, which is consistent with the findings mentioned earlier. Many other clinical studies also have confirmed that SGLT2 inhibitors are widely used to treat HF patients due to their clinical benefits.^[16]^ There are several potential mechanisms about how SGLT2 inhibitors may reduce LV dilation and improve cardiac function for patients with HF and DCM. Previous experimental and clinical data have shown that SGLT2 inhibitors cause a reduction in preload by promoting natriuresis and diuresis, thus the consequent reduction in cardiac dilation and remodeling.^[17]^ Moreover, in the nondiabetic porcine model of HF, it has been found that SGLT2 inhibitors significantly ameliorated adverse anatomical LV remodeling and enhanced LV systolic function. These cardiac benefits of SGLT2 inhibitors are mediated by a switch in myocardial fuel metabolism away from glucose metabolism toward ketone bodies, free fatty acid, and branched-chain amino acid, which increased myocardial adenosine triphosphate content and enhanced myocardial work efficiency.^[18]^ Shao et al reported that the SGLT2 inhibitor empagliflozin can ameliorate cardiac structural as well as improve mitochondrial biogenesis and function.^[19]^ Thus, SGLT2 inhibitors might adverse LV remodeling by enhancing myocardial energetics in DCM patients.

In addition, the accumulating evidence points to an essential role of the inflammatory component in the process of DCM.^[20,21]^ Recent studies suggest that SGLT2 inhibitors may have anti-inflammatory effects. A mouse DCM model was used to demonstrate that dapagliflozin prevents ventricular dilatation and improves cardiac function by reducing toll-like receptor 4 expression and inhibiting nucleotide-binding oligomerization domain-like receptor family protein 3 inflammasome pathway activation.^[12]^ In human tubular epithelial cells, SGLT2 inhibitors remarkably reversed glucose-induced reducing toll-like receptor 4expression.^[22]^ So, we speculated that SGLT2 inhibitors reduce cardiac dilation and remodeling and improve cardiac function in DCM patients through its anti-inflammatory effects. Chronic activation of neurohormonal response, especially of the sympathetic nervous system and natriuretic peptides, is a major hallmark of adverse LV remodeling.^[23]^ Experimental and clinical studies indicate that SGLT2 inhibitors reduced plasma levels of normetanephrine and natriuretic peptides, suggesting an interruption of the pathological neurohormonal activity.^[24–26]^

Taken together, SGLT2 inhibitors have been shown to promote diuresis, as well as reduce inflammasomes and sympathetic overdrive, all of which play important roles in fostering DCM. SGLT2 inhibitors reduce LV dilation and improve cardiac function for patients with DCM may by protective effects through the aforementioned several potential mechanisms.

## 5. Limitations

Several limitations associated with the present study warrant mention. In small single-center clinical studies like this, imbalances in baseline characteristics (such as HbA1c and glucose) may occur, potentially influencing the interpretation of randomized trial effects. Additionally, the sample size is too small and predominantly consists of male participants. Thus, further studies with larger patient populations will be needed to validate our findings. The effects of SGLT2 inhibitors in NIDCM patients were observed at 12 months of follow-up. Longer follow-up will also help determine the effect of SGLT2 inhibitors on reduction of LV dilation and improvement of LV function in NIDCM patients. What is more, NIDCM encompasses a wide range of etiologies, which can be broadly categorized as either primary or genetic, and secondary or non-genetic, including disorders such as valvular heart disease, inflammatory myocardial disease, or the toxic effects of drugs, or chemotherapy agents.^[27]^ This clinical study investigates the various etiologies and their combinations in patients with NIDCM, without further differentiation of the underlying causes.

## 6. Conclusion

Although previous studies have demonstrated the significant efficacy of SGLT2 inhibitors in improving left ventricular remodeling and systolic function among HF patients, there is a paucity of clinical research investigating the application of these inhibitors in individuals with NIDCM. Our study provides evidence for the clinical application of SGLT2 inhibitors in reducing LV dilation and improving LV function in NIDCM patients. Given the improvement in LV structure and function with SGLT2 inhibitors, it is indicated that SGLT2 inhibitors can further reduce LV dilation and improve cardiac function even when added to the background of current standard therapies in NIDCM patients.

## Acknowledgments

We would like to acknowledge the hard and dedicated work of all the staff of the study.

## Author contributions

**Formal analysis:** Nake Jin.

**Methodology:** Xuechen Zhao.

**Project administration:** Lei Huang.

**Software:** Nake Jin, Jianan Hu.

**Writing – original draft:** Jun Hong.

**Writing – review & editing:** Lei Huang.

## Supplementary Material


