# Supplementary material for: Effect of dapagliflozin on left ventricular structure and function in patients with non-ischemic dilated cardiomyopathy: An observational study
Source: Medicine (Baltimore). 2024 Mar 29;103(13):e37579. doi: 10.1097/MD.0000000000037579 (PMC10977548; doi:10.1097/MD.0000000000037579)
Supplement: Supplementary file 1 [file medi-103-e37579-s001.docx]

Supplementary Table 1 Results of MANCOVA for the change of echocardiographic parameters

|  | F | p |
| --- | --- | --- |
| SGLT2 inhibitors | 4.649 | 0.001 |
| [gender](javascript:;) | 1.023 | 0.438 |
| Age | 3.436 | 0.005 |
| BMI | 1.232 | 0.311 |
| Heart rate | 0.523 | 0.831 |
| Systolic blood pressure | 0.598 | 0.773 |
| Diastolic blood pressure | 0.931 | 0.517 |

SGLT2 sodium glucose cotransporter type 2,BMI Body Mass Index
